# Differences in quality of anticoagulation care delivery according to ethnoracial group in the United States: A scoping review

**DOI:** 10.1007/s11239-024-02991-2

**Published:** 2024-05-11

**Authors:** Sara R. Vazquez, Naomi Y. Yates, Craig J. Beavers, Darren M. Triller, Mary M. McFarland

**Affiliations:** 1 University of Utah Health Thrombosis Service, 6056 Fashion Square Drive, Suite 1200, Murray, UT 84107 USA; 2 Kaiser Permanente Clinical Pharmacy Services, 200 Crescent Center Pkwy, Tucker, GA 30084 USA; 3 Anticoagulation Forum, Inc, 17 Lincoln Street, Suite 2B, Newton, MA 02461 USA; 4 University of Kentucky College of Pharmacy, 789 S Limestone, Lexington, KY 40508 USA; 5 University of Utah Spencer S. Eccles Health Sciences Library, 10 N 1900 E, Salt Lake City, UT 84112 USA

**Keywords:** Racial, Ethnoracial, Disparities, Anticoagulation, Quality

## Abstract

Anticoagulation therapy is standard for conditions like atrial fibrillation, venous thromboembolism, and valvular heart disease, yet it is unclear if there are ethnoracial disparities in its quality and delivery in the United States. For this scoping review, electronic databases were searched for publications between January 1, 2011 – March 30, 2022. Eligible studies included all study designs, any setting within the United States, patients prescribed anticoagulation for any indication, outcomes reported for ≥ 2 distinct ethnoracial groups. The following four research questions were explored: Do ethnoracial differences exist in 1) access to guideline-based anticoagulation therapy, 2) quality of anticoagulation therapy management, 3) clinical outcomes related to anticoagulation care, 4) humanistic/educational outcomes related to anticoagulation therapy. A total of 5374 studies were screened, 570 studies received full-text review, and 96 studies were analyzed. The largest mapped focus was patients’ access to guideline-based anticoagulation therapy (88/96 articles, 91.7%). Seventy-eight articles made statistical outcomes comparisons among ethnoracial groups. Across all four research questions, 79 articles demonstrated favorable outcomes for White patients compared to non-White patients, 38 articles showed no difference between White and non-White groups, and 8 favored non-White groups (the total exceeds the 78 articles with statistical outcomes as many articles reported multiple outcomes). Disparities disadvantaging non-White patients were most pronounced in access to guideline-based anticoagulation therapy (43/66 articles analyzed) and quality of anticoagulation management (19/21 articles analyzed). Although treatment guidelines do not differentiate anticoagulant therapy by ethnoracial group, this scoping review found consistently favorable outcomes for White patients over non-White patients in the domains of access to anticoagulation therapy for guideline-based indications and quality of anticoagulation therapy management. No differences among groups were noted in clinical outcomes, and very few studies assessed humanistic or educational outcomes.

**Graphical Abstract:**

Scoping Review: Differences in quality of United States anticoagulation care delivery by ethnoracial group. AF = atrial fibrillation; AMS = anticoagulation management service; DOACs = direct oral anticoagulants; INR = international normalized ratio; PSM = patient self-management; PST = patient self-testing

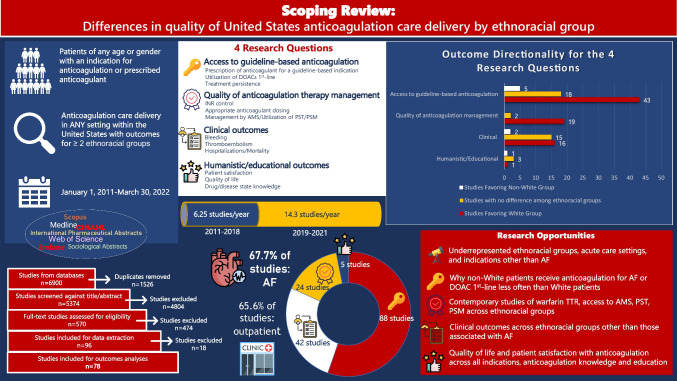

**Supplementary Information:**

The online version contains supplementary material available at 10.1007/s11239-024-02991-2.

## Introduction

It is well-established that in the United States (US) ethnoracial disparities exist in various aspects of health care. Specifically, persons identifying with an ethnoracial minority group may have more challenging access to health care, worse clinical outcomes, and higher dissatisfaction with care compared to White persons [1–5]. There are differences by ethnoracial group in the prevalence of the three most common indications for which anticoagulants are prescribed, stroke prevention in atrial fibrillation (AF), treatment of venous thromboembolism (VTE), and valvular heart disease [6–17]. Specifically, VTE is most prevalent in Black patients compared to White and Asian patients, whereas AF is most prevalent in White patients compared to Black, Asian, and Hispanic patients [9, 10, 15]. Calcific heart valve disease has the most relevance to the US population, and epidemiologic data has shown that aortic stenosis is more prevalent in White patients compared to Black, Asian, and Hispanic patients [17]. Despite these epidemiologic differences, there is no evidence to suggest there should be any difference in treatment strategies across ethnoracial patient groups.

While studies have demonstrated genotypic differences that may result in different warfarin dose requirements[18], and early studies may indicate genotypic differences in direct oral anticoagulant (DOAC) response [19], no US-based labeling or guidelines recommend a difference in prescription or delivery of anticoagulation care based on race or ethnicity. However, it is unclear if there are in fact differences in the type and quality of anticoagulation therapy, which is standard of care for each of these conditions [20–24]. Anticoagulants remain in the top three classes of drugs causing adverse drug events (primarily bleeding) in the United States, according to the 2014 National Action Plan for Adverse Drug Event Prevention. One of the goals of the National Action Plan was to identify patient populations at higher risk for these adverse drug events to inform the development of targeted harm reduction strategies [25]. If ethnoracial minority patients are receiving sub-optimal anticoagulation therapy in certain measurable areas of anticoagulation quality, it is vital to highlight the areas of disparity so that these can be explored and care optimized. Anticoagulation providers often have high frequency contact with their patients and can be a reliable connection between disproportionately affected patients and a system in need of change. Systematic reviews of ethnoracial disparities in AF and VTE have been conducted. The AF review assessed AF prevalence among racial groups as well as differences in symptoms and management, including stroke prevention with warfarin or DOACs [9]. The VTE review specifically assessed VTE prevalence and racial differences in COVID-19 and did report the use of any prophylactic anticoagulation, but this was not part of the analysis [26]. No review of racial disparities in quality of anticoagulation therapy was found in search results conducted prior to protocol.

In this study we aimed to identify any potential ethnoracial disparities in anticoagulation care quality in the US. The decision to limit the study to a US population was based on our observation that the US has a unique history of interactions between racial and ethnic groups that may not necessarily be reflected by studies conducted in other countries. Additionally, health care delivery systems vary widely across the world, and we wanted to include the data most relevant to the potential racial disparities existing in the US health care system. The term “race” was used to identify a group of people with shared physical characteristics believed to be of common ancestry whereas the term “ethnicity” refers to a group of people with shared cultural traditions [27]. We recognize these terms may be far more complex. In order to encompass both the physical and cultural aspects of a patient’s identity we have chosen to use the term “ethnoracial” for this study [27]. Highlighting existing differences will serve as a stimulus for institutions and clinicians to assess current services, implement quality improvement measures, and inform future research efforts to deliver optimal anticoagulation care for all patients. The scoping review protocol was registered December 22, 2021 to Open Science Framework, 10.17605/OSF.IO/9SE7H [28].

## Methods

We conducted this scoping review with guidance from the 2020 version of the *JBI Manual for Evidence Synthesis* and organized to Arksey's five stages: 1) identifying the research question, 2) identifying relevant studies, 3) study selection, 4) charting the data and 5) collating, summarizing and reporting the results [29, 30]. For transparency and reproducibility, we followed the PRISMA-ScR and PRISMA-S reporting guidelines in reporting our results [31]. We used Covidence (Veritas Health Innovation,) an online systematic reviewing platform to screen and select studies. Citation management and duplicate detection and removal was accomplished with EndNote, version 19 (Clarivate Analytics.) Data was charted from our selected studies using REDCap, an electronic data capture tool hosted at the University of Utah [32].

### Literature searching

An information specialist developed and translated search strategies for the online databases using a combination of keywords and controlled subject headings unique to each database along with team feedback. Peer review of the strategies was conducted by library colleagues using the PRESS guidelines. [33] Electronic databases searched included Medline (Ovid) 2011–2022, Embase (embase.com) 2011–2022, CINAHL Complete (Ebscohost) 2011–2022, Sociological Abstracts (ProQuest) 2011–2022, International Pharmaceutical Abstracts (Ovid) 2011–2022, Scopus (scopus.org) 2011–2022 and Web of Science Core Collection (Clarivate Analytics) 2011–2022. Limits included a date range from January 1, 2011 to March 30—April 19, 2022, as not all database results were exported on the same day. See Supplemental File 1 for detailed search strategies. A search of grey literature was not conducted due to time and resource constraints.

### Study Selection

For inclusion, each study required two votes by independent reviewers for screening of titles and abstracts followed by full-text review. A third reviewer provided the deciding vote. Data extraction was performed by two independent reviewers, and consensus on any discrepancies was reached via discussion between the reviewers. The data form was piloted by two team members using sentinel articles prior to data extraction.

Eligible studies included all types of study designs in any setting with a population of patients of any age or gender located within the US who were prescribed anticoagulant therapy for any indication, published between January 1, 2011 – March 30, 2022 in order to capture contemporary and clinically relevant practices.

We defined the following research questions for this scoping review as described in Table 1.

**Table 1:** Research Questions: Do ethnoracial differences exist in the following areas of anticoagulation care delivery?

**Research Question** **1**: Access to guideline-based anticoagulation therapy Prescription of anticoagulant medication for a guideline-based indication (i.e., stroke prevention in AF based on a risk score) Utilization of direct oral anticoagulants (DOACs) (apixaban, dabigatran, edoxaban, rivaroxaban) as first-line therapy for AF or VTE* Anticoagulant therapy adherence/persistence
**Research Question** **2**: Quality of anticoagulation therapy management Warfarin INR control Appropriate anticoagulant dosing (i.e., DOAC) Management by an anticoagulation management service Utilization of PST/ PSM
**Research Question** **3**: Clinical outcomes related to anticoagulation care Bleeding Thromboembolism Hospitalizations All-cause mortality
**Research Question** **4**: Humanistic/Educational outcomes related to anticoagulation therapy Patient satisfaction with anticoagulation therapy Quality of life related to anticoagulation therapy or its indication Educational delivery models Knowledge assessment

AF = atrial fibrillation; DOAC = direct oral anticoagulant; INR = International Normalized Ratio; PSM = patient self-management; PST = patient self-testing; VTE = venous thromboembolism.

*Direct oral anticoagulants were recommended as first-line therapy as early as 2016 for venous thromboembolism treatment (American College of Chest Physicians 2016)[34] and 2018 for atrial fibrillation (American College of Chest Physcians 2018) [35]

Studies must have reported any of these anticoagulation care delivery outcomes for at least 2 distinct racial or ethnic groups. We excluded genotyping studies and non-English language articles at full text review, as we had no funding for translation services. In checking references of included studies, no additional studies met inclusion criteria. In accordance with scoping review methodology, no quality assessment of included studies was conducted as our goal was to rapidly map the literature. As this is a scoping review of the literature, no aggregate or pooled analysis was performed; however, for ease of interpretation, when assessing for the directionality of the outcomes in the various studies, we categorized studies into Favoring White Group, Favoring Non-White Group, and No Differences Among Ethnoracial Groups. If studies had mixed outcomes of favoring one group for one outcome and no difference for another, then the study was categorized with the favoring group.

## Results

**Fig. 1:**
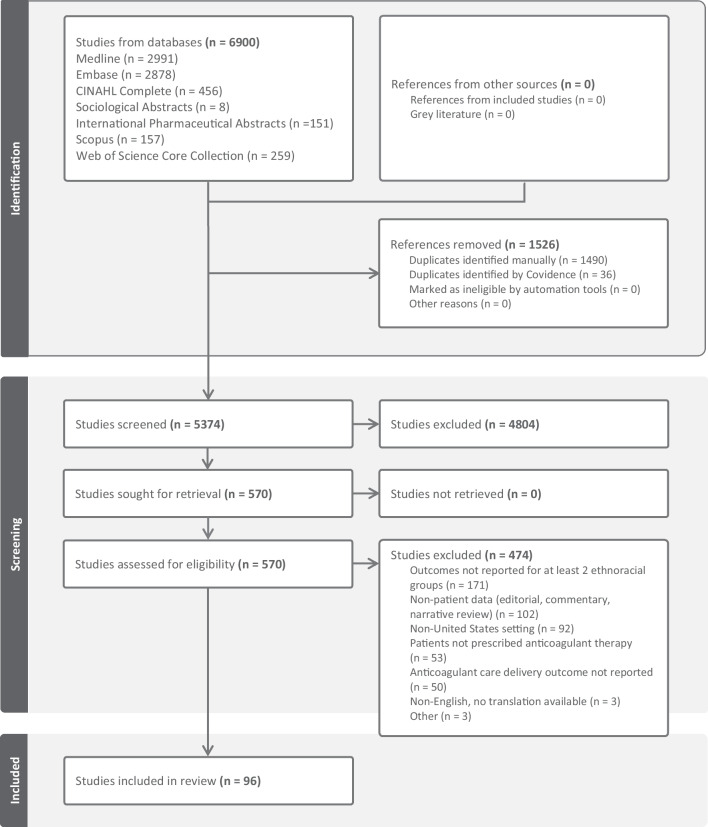
PRISMA Flow Diagram

A PRISMA flow diagram in Fig. 1 depicts search results, exclusions, and inclusions. The search strategies retrieved 6900 results with 1526 duplicates removed. Following title and abstract screening of 5374 references, 570 articles received full-text review. The most common reason for the exclusion of 474 studies was that outcomes were not reported for two distinct ethnoracial groups (171 studies). Ninety-six studies underwent data extraction.

### Study characteristics-overall

**Fig. 2:**
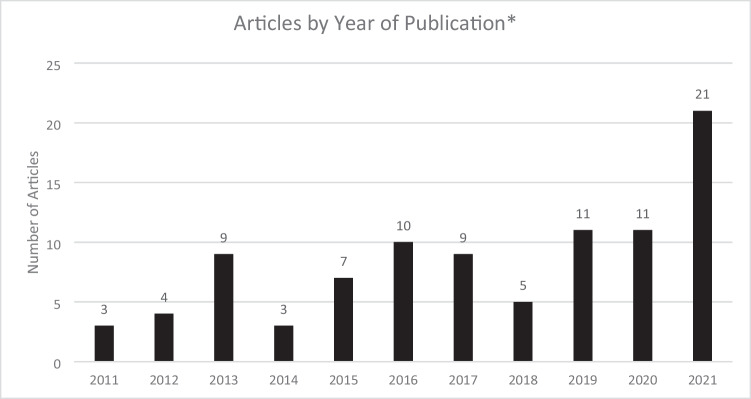
Number of Articles by Publication Year. *2022 excluded from this figure since the search period did not capture the entire year

Fifty of the 96 studies were published between 2011 and 2018 (an average of 6.25 articles per year that compared outcomes between two ethnoracial groups) and 43 of 96 studies were published in the years 2019–2021 (average 14.3 articles per year; 2022 excluded here because only 4 months of data was captured) (Fig. 2). Most studies analyzed an outpatient population (65.6%) for an indication of stroke prevention in AF (67.7%) in patients taking warfarin (71.9%) or DOACs (49.0%). Study population size was heterogenous, ranging from a study size of 24 patients to over 1.3 million patients (median 5,238 patients) in the 69 studies that reported population size by racial group. When stratified by size, 60.9% of the articles in the scoping review (42 articles) represented < 10,000 patients (Table 2).

**Table 2:** Study Characteristics

Study Characteristic	Number of articles (%)
*Study Design*	
Retrospective cohort	59 (61.5)
Registry	12 (12.5)
Other	10 (10.4)
Prospective cohort	7 (7.3)
Randomized controlled trial	5 (5.2)
Systematic review*	3 (3.1)
*Study Magnitude*	
Multi-center	39 (40.6)
Claims database	29 (30.2)
Single-center	23 (24.0)
Other	5 (5.2)
Study Population Size**	
< 1000 patients	22 (31.9)
1001–10,000 patients	20 (29.0)
10,001–100,000 patients	18 (26.1)
> 100,000 patients	9 (13.0)
Region of the United States***	
Unspecified/Other or Nationwide	54 (56.3)
Northeast	26 (27.1)
Midwest	19 (19.8)
West	14 (14.6)
Southeast	10 (10.4)
Southwest	5 (5.2)
Puerto Rico/Other US Territories	4 (4.2)
Study Setting***	
Outpatient	63 (65.6)
Inpatient	33 (34.4)
Not specified	8 (8.3)
Emergency department	6 (6.3)
Non-acute care facility	3 (3.1)
Indication for Anticoagulation***	
Atrial fibrillation	65 (67.7)
VTE treatment	19 (19.8)
Not specified	11 (11.5)
Stroke	8 (8.3)
VTE prophylaxis	5 (5.2)
Mechanical heart valve	3 (3.1)
Anticoagulant Studied***	
Warfarin	69 (71.9)
Any DOAC	47 (49.0)
Any injectable anticoagulant	12 (12.5)
Any oral anticoagulant	10 (10.4)
Any anticoagulant	5 (5.2)
Not specified	3

DOAC = direct oral anticoagulant; US = United States; VTE = venous thromboembolism

*As the purpose of the scoping review was to map the literature, we did include systematic reviews. Six total studies were represented in our search and in the systematic reviews

**Only 69 of the total 96 studies reported study population size by racial group

***multiple categories may apply, therefore total may exceed 100%

### Study characteristics-by ethnoracial group

**Fig. 3:**
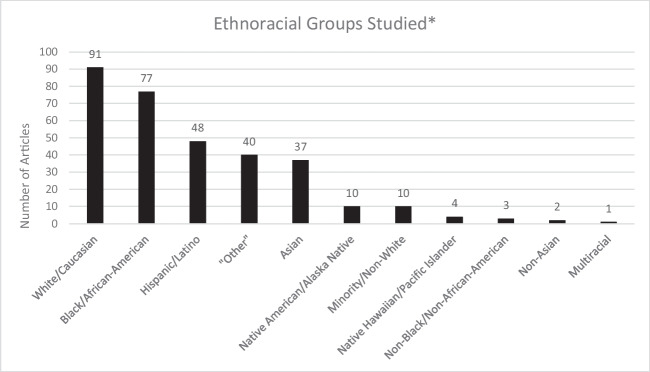
Number of Articles by Ethnoracial Groups. *For study inclusion, a study had to compare outcomes for least two distinct ethnoracial groups

There were 50 studies (52.1%) where race or ethnicity was either mentioned in the title or objective of the article, with 24 of these published over the 7-year period 2011–2018 and 26 published over the 3-year period 2019 to first quarter 2022. The method for reporting race or ethnicity was unclear or unspecified in most studies (77.1%) and 16 articles (16.7%) utilized self-reporting of race or ethnicity. Most studies analyzed White or Caucasian racial groups (94.8%), followed by Black or African-American (80.2%), and many studies grouped all other racial groups into an “Other” category (41.7%) (Fig. 3).

White patients accounted for a median 77% of study populations, Black patients 9.5%, Hispanic/Latino patients 6.2%, “Other” racial groups 5.3%, and Asian patients 2.5%.

### Study outcomes-overall

**Fig. 4:**
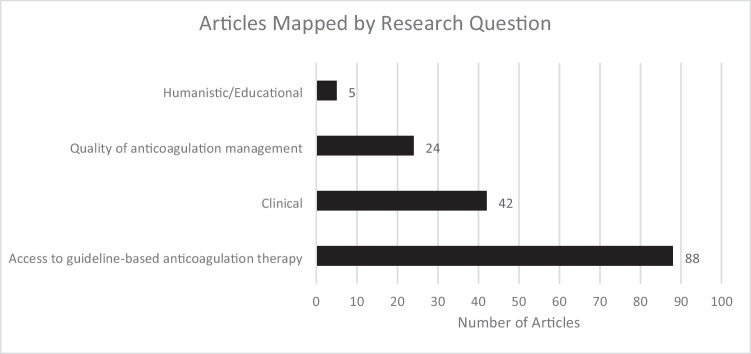
Number of Articles Mapped by Research Question

Of the 4 research questions, most studies included in this review analyzed patients’ access to guideline-based anticoagulation therapy (88/96 articles, 91.7%), clinical outcomes (42/96 articles, 43.8%), or quality of anticoagulation management (24/96 articles, 25.0%), while very few addressed humanistic or educational outcomes (5/96 articles, 5.2%) (Fig. 4). Many studies addressed multiple outcomes within the single study.

Seventy-eight of the 96 included studies provided statistical comparisons between ethnoracial groups, and these data are presented below.

### Outcomes for research question 1: Do ethnoracial differences exist in access to guideline-based anticoagulation therapy?

## Anticoagulation for a guideline-based indication

This question focused on patients who had an indication for anticoagulation actually receiving an anticoagulant, specifically AF and VTE prophylaxis (based on risk stratification) and acute VTE. The majority of the AF studies (25/34 studies) demonstrated White patients receiving anticoagulation at significantly higher rates compared to non-White patients [36–60], while the six VTE studies largely demonstrated no difference among ethnoracial groups [61–66].

## DOACs as first-line therapy for AF or VTE

Eighteen individual studies statistically assessed the outcome of DOAC as first-line therapy (compared to warfarin) for AF (15 studies), VTE treatment (2 studies), or both indications (1 study). Twelve of the 15 AF studies showed a significantly higher proportion of White patients received DOACs as first-line therapy compared to non-White patients [36, 40–46, 54, 55, 67, 68]. Of those 12, 9 specifically compared White patients to Black patients. Both VTE treatment studies and the study that assessed both AF and VTE indications showed significantly higher DOAC prescribing rates for White patients compared to Black patients [69–71].

## Anticoagulant therapy adherence/persistence

The eight studies that addressed anticoagulation therapy adherence/persistence showed variability in outcome directionality by ethnoracial group: 5 no difference [41, 72–75], 2 showed better treatment adherence/persistence for White patients compared to Black patients[76] or non-White patients [77], and one showed better treatment adherence/persistence for White patients compared to Hispanic patients, but no difference in White versus Black patients [78].

**Fig. 5:**
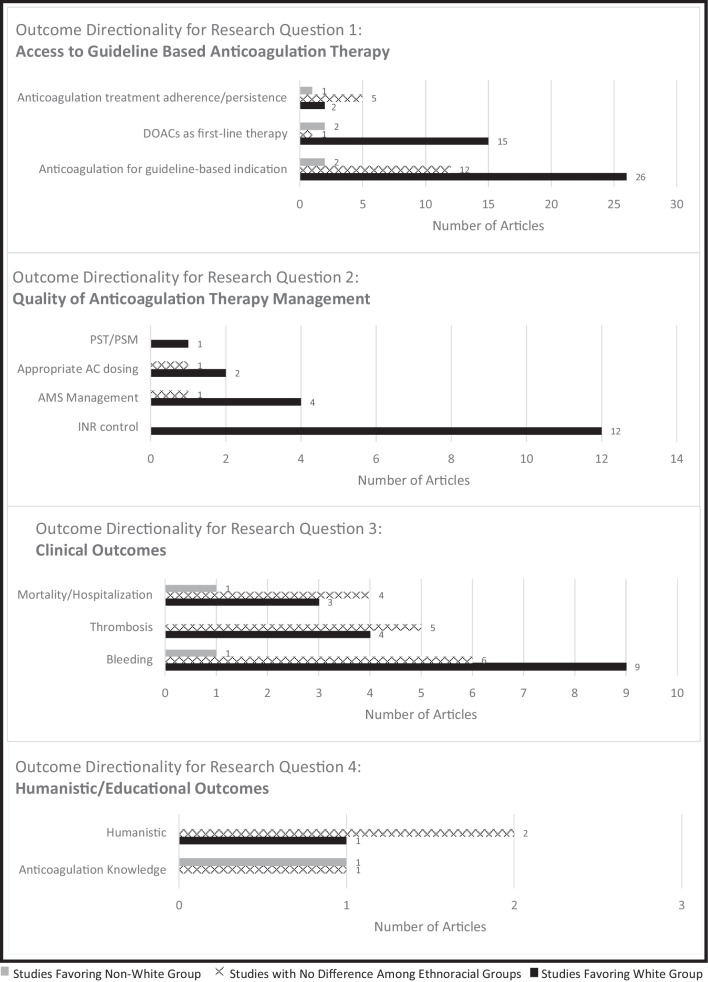
Outcome Directionality for the 4 Research Questions and their Subcategories. AC = anticoagulant; AMS = anticoagulation management service; INR = international normalized ratio; PST = patient self-testing; PSM = patient self-management

Figure 5 summarizes the outcome directionality for Research Question 1 regarding access to guideline-based anticoagulation therapy. Overall, the areas of disparity identified included anticoagulation for atrial fibrillation and preferential use of DOAC therapy for AF and VTE treatment.

### Research question 2: Do ethnoracial differences exist in the quality of anticoagulation therapy management?

A total of 21 studies assessed quality of anticoagulation therapy management: Warfarin time in therapeutic range (TTR)/INR (International Normalized Ratio) control 12 studies, appropriate anticoagulant dosing 3 studies, enrollment in an anticoagulation management service 5 studies, and PST/PSM one study.

In statistical comparisons of INR control in warfarin patients, all 12 studies (7 assessed mean or median TTR, 5 assessed other measures of INR control such as days spent above/below range, gaps in INR monitoring) showed White patients had favorable INR control compared to non-White patients (most comparisons included Black patients) [41, 75, 79–87]. Enrollment in an anticoagulation management service was statistically compared among ethnoracial groups in 5 studies, and this opportunity favored White patients compared to other racial groups in four of the five [41, 82, 86, 88]. Two of the three studies that statistically analyzed appropriate anticoagulant dosing showed a higher rate of appropriate DOAC dosing in White patients compared to non-White patients [41, 89], and the third showed no difference among ethnoracial groups for enoxaparin dosing in the emergency department [90]. The one study assessing access to PST/PSM showed that more White patients used PST compared to Black or Hispanic patients[91] (Fig. 5).

### Research question 3: Do ethnoracial differences exist in the clinical outcomes related to anticoagulation care?

Articles assessing clinical outcomes among ethnoracial groups primarily assessed bleeding (15 articles) or thrombosis (9 articles) outcomes, and 8 articles assessing anticoagulation related hospitalization or mortality. One article addressed a net clinical outcome including major bleeding, stroke or systemic embolism, and death from any cause. This was included in the bleeding outcomes category so that it was not double-counted in the other two outcome categories. Additional details about the 24 unique studies that statistically assessed clinical outcomes including the study design, population size, ethnoracial groups studied, anticoagulants used, and statistical outcomes measured can be found in Supplementary Tables 1 and 2.

Sixteen studies statistically assessed bleeding outcomes of varying definitions (major bleeding 13 studies, clinically relevant non-major bleeding 3 studies, any bleeding 3 studies, bleeding otherwise defined 3 studies). Six studies demonstrated no difference in bleeding outcomes by ethnoracial group [55, 92–96]9 reported that White patients had lower rates of bleeding compared to Black or Asian patients,[53, 80, 83, 85, 97–101]. In the remaining study, Asian patients had a more favorable net clinical outcome compared to non-Asian patients [102].

Nine studies statistically assessed thrombosis outcomes among ethnoracial groups, including stroke/systemic embolism (5 studies), recurrent VTE (3 studies), or any thrombosis (1 study). The stroke outcomes by racial group were heterogeneous, with 3 studies showing better outcomes for White patients compared to Black patients[103–105] and two studies showing no difference in outcomes when White patients were compared to Non-White patients [55, 95]. In three of the four VTE studies there were no differences in outcomes by ethnoracial group [61, 93, 96], and in one study White patients had more favorable outcomes compared to Black patients [106].

Nine studies assessed anticoagulation-related hospitalizations or mortality by ethnoracial group. Outcomes were mixed, as four studies showed no difference in hospitalizations or mortality among ethnoracial groups,[89, 95, 96, 107], three studies showed White patients had a lower rate of hospitalizations[85, 105] or mortality[104, 105] Another study showed lower rate of mortality or hospice after intracranial hemorrhage in Black and Other race patients [108].(Fig. 5).

### Research question 4: Do ethnoracial differences exist in the humanistic/educational outcomes related to anticoagulation therapy?

The five studies reporting this category of outcomes were heterogeneous. Of the two studies assessing anticoagulation knowledge, one showed no difference by ethnoracial group [109], and the other favored the non-White group in appropriately estimating bleeding risk [110]. One study assessed an atrial fibrillation quality of life score at 2-year follow-up after AF diagnosis and found the outcomes favored White patients [79]. Another study assessed satisfaction with VTE care and found no difference among ethnoracial groups [111]. A third study found no difference in the percentage of racial groups having a cost conversation when initiating DOAC therapy (78% Whites, 72.2% non-Whites)[112] (Fig. 5).

**Fig. 6:**
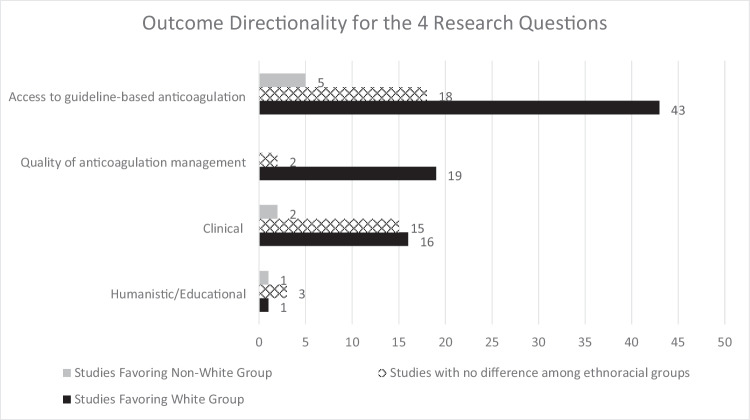
Outcome Directionality for All 4 Research Questions

Overall outcome directionality for all four research questions is shown in Fig. 6. A total of 79 articles demonstrated favorable outcomes for White patients compared to non-White patients, 38 articles showed no difference between White and non-White groups, and 8 articles had outcomes favoring non-White groups (the total exceeds the 78 articles with statistical outcomes as many articles reported multiple outcomes). The biggest areas of disparity between White and non-White groups are access to guideline-based anticoagulation therapy and quality of anticoagulation therapy management. Clinical outcomes relating to anticoagulation care had the least difference among ethnoracial groups. Relatively few studies assessed potential ethnoracial disparities in humanistic and educational outcomes.

## Discussion

This scoping review assessing ethnoracial differences in the quality of anticoagulation care and its delivery to patients in the United States encompassed eleven full years of literature and resulted in the inclusion of 96 studies, 78 of which contained statistical outcomes comparisons among ethnoracial groups. The most common reason for study exclusion was that outcomes were not reported for at least two distinct ethnoracial groups. We observed that beginning in 2019 and following the racial unrest of 2020, the density of articles addressing ethnoracial disparities in anticoagulation care more than doubled. During the entire study period, half of studies had race or ethnicity as the focus or objective of the paper, but this was largely driven by articles published after 2019.

Only 16% of included articles documented self-reporting of racial identity, with most of the remainder using an unspecified method for documenting racial identity. It is likely that many studies utilize demographic information extracted from an electronic medical record (EMR), but it is often unclear if that is truly self-reported race. A second element this scoping review identified was that many studies analyzed two or three ethnoracial groups and then categorized all others into a heterogenous “Other” category. For example, frequently studies would categorize patients as White, Black, and “Other.” It is unclear whether those in a racial category labeled as “Other” had an unknown or missing racial identity in the EMR, or intentionally chose not to disclose. It is also likely that study investigators decided to classify ethnoracial groups with lower population sizes into a miscellaneous category. There were few studies (15%) that specifically assessed patients identifying as Native American/Alaska Native, Native Hawaiian/Pacific Islander, and multiracial. While Hispanic/Latino is an ethnicity, most studies categorized it as a separate “race” category. Of the 37 studies that analyzed “Asian” patient populations, none specifically defined “Asian” beyond that. The US Census Bureau defines “Asian” race as a person having origins of the Far East, Southeast Asia, or the Indian subcontinent [113]. This broad definition encompasses many different ethnicities which could represent variability in health outcomes if better defined and more frequently analyzed. These may be opportunities for EMR systems to improve transparency for how race, ethnicity, and language preference are captured and for those designing research studies to be thoughtful and intentional about analyzing the ethnoracial identities of the study population, perhaps in alignment with the minimum 5 racial categories utilized by the US Census Bureau, the National Institutes of Health, and the Office of Management and Budget (White, Black, American Indian/Alaska Native, Asian, Native Hawaiian/Pacific Islander, with permission for a “some other race” category and the option to select multiple races) [113]. Since 2017 Clinicaltrials.gov has required the reporting of race/ethnicity if collected, and there is good compliance with this requirement, but less so in publication of the work [114].

We examined the proportion of ethnoracial groups represented for each of the disease states in the studies included in this scoping review, relative to disease state prevalence and found a discrepancy. For AF, prevalence in White patients was 11.3%, in Black patients 6.6%, and in Hispanic patients 7.8% [15]. However, the representation in AF studies in this review were 74% White, 13% Black, and 8% Hispanic. Assessing VTE incidence by race is more difficult, as studies have shown regional and time variation, with Black patients typically having a higher incidence compared to other ethnoracial groups [16]. In this review, however, of the studies assessing VTE treatment or prophylaxis, only 16% of the patient population identified as Black, whereas 70% identified as White. There were only 3 studies that assessed a valvular heart disease population, making ethnoracial group representation difficult to assess.

The majority of studies captured in this review analyzed patients in the outpatient setting, for the anticoagulation indication of stroke prevention in AF, taking either warfarin or DOAC. Few studies involved the acute care setting or injectable anticoagulants, representing an area for future study of potential ethnoracial disparities.

Overall, the majority of studies in this scoping review addressed ethnoracial disparities in patients’ access to guideline-based anticoagulation therapy, clinical outcomes related to anticoagulation care, and quality of anticoagulation management. A research gap identified was more study is needed to assess gaps in educational outcomes such as anticoagulation and disease state knowledge, shared decision-making willingness and capability, and humanistic outcomes such as quality of life or satisfaction with anticoagulation therapy.

In analyzing the first research question regarding ethnoracial differences in access to guideline-based anticoagulation therapy, the majority of studies addressed use of any anticoagulation for stroke prevention in AF in patients above a threshold risk score and the preferential use of DOACs as first-line therapy instead of warfarin for AF. In both categories, patients in a non-White ethnoracial group (particularly Black patients) received recommended therapy less often than patients identified as White. It is unclear why this is the case. It could be on the patient, provider, and/or system level. It is possible that some studies more successfully adjusted for covariates than others. Sites or settings with systematic processes like order sets or clinical decision support systems in place for standard prescribing may be more successful in equitably prescribing indicated therapies. In one large study in the Veterans Affairs population of AF patients, even after adjusting for numerous variables that included clinical, demographic, socioeconomic, prescriber, and geographic site factors, DOAC prescribing remained lower in Asian and Black patients when compared with White patients. The authors in that study postulate that non-White populations may be less receptive to novel therapies due to historical mistrust of the health care system or have reduced access to education about the latest treatments, and they give the example of direct-to-consumer advertising [42]. It has also previously been demonstrated that prescribing of oral anticoagulation and particularly DOACs is lower in non-White patients [41]. These are difficult to capture as standard covariates, which is why further study is needed. We examined the publication dates for both access categories to see if perhaps there was a lack of contemporary data skewing the outcomes. However, for both anticoagulation for a guideline-based indication and DOACs as first-line therapy, the majority of articles came from the time period 2019–2021 (24 of 40 articles, and 15 of 18 articles, respectively), well after guideline updates preferentially recommended DOACs [34, 35]. Also, there were relatively few studies addressing guideline-based therapy for VTE treatment and prophylaxis, making assessment of disparities difficult. Regarding access, it is well established that race and ethnicity often determine a patient’s socioeconomic status and that low socioeconomic status and its correlates (e.g., reduced education, income, and healthcare access) are associated with poorer health outcomes [115]. However, at each level of income or education, Black patients experience worse health outcomes than Whites [116]. So, low socioeconomic status does not fully explain poorer health outcomes for non-White individuals.

After examining access to appropriate and preferred anticoagulation therapy, the second research question of this scoping review examined potential ethnoracial disparities in the quality of anticoagulation therapy management. INR control measures such as time in therapeutic INR range are a surrogate measure of both thrombotic and bleeding outcomes and frequently used as a way to assess quality of warfarin therapy. The studies identified in this review showed clear disparity between White and non-White patient groups (especially Black patients), however *all* twelve studies comparing TTR among ethnoracial groups were published prior to 2019. This could be due to the decline in warfarin prescribing relative to increases in DOAC prescribing [117–119], but there remain patient populations that require or choose warfarin, so this marker of anticoagulation control remains relevant and requires continued reassessment. There were relatively few studies assessing other markers of anticoagulation management quality such as anticoagulation management service enrollment, appropriate DOAC dosing, and access to quality improvement strategies like PST or PSM. Few studies assessed educational outcomes, yet this may have relevance to the above anticoagulation care quality question. For those patients who remain on warfarin, dietary Vitamin K consistency is an example of a key educational point that links directly to INR control. It is unclear if there are disparities in this type of education among ethnoracial groups that may have more far-reaching effects.

Of note, clinical outcomes related to anticoagulant therapy seemed to have the fewest areas of disparity, although the number of articles was small. This suggests that if patients have access to high quality anticoagulation therapy, there is a promising sign that optimal clinical outcomes can be achieved for all ethnoracial groups.

There are some limitations of this scoping review that warrant consideration. First, we chose fairly broad inclusion criteria (all anticoagulants, all study types) because a review of this type had never been performed before. This resulted in a relatively large number of included articles for a scoping review. Second, there is likely a high degree of heterogeneity among patient populations and outcomes definitions. However, as this is a scoping review with the goal to present an overview of the literature and not report on composite outcomes, a risk of bias assessment was not performed. Third is our decision to group patients into White and non-White groups for assessment of outcome directionality. In doing so, we may have missed subtle differences in outcomes between various non-White ethnoracial groups. Fourth, in our main search we included all studies that reported outcomes, but due to scope, we only reported outcome directionality for studies that statistically compared outcomes between ethnoracial groups. Finally, due to the large number of studies that required review and analysis, this was a lengthy undertaking and we are certain that additional studies have been published since the closure of our search period.

In line with the 2014 National Action Plan for Adverse Drug Event Prevention’s goal of identifying patient populations at higher risk of adverse drug events, this scoping review highlights several areas where quality of anticoagulation care can be optimized for all patients. Future research opportunities in ethnoracial differences in the quality of anticoagulation care are summarized in Table 3. While the scoping review focused exclusively on the evaluation of peer-reviewed manuscripts, the heterogeneity of terminology and methodologies identified in the published papers may have implications for national health policy relating to the quality and safety of care (e.g.the Medicare Quality Payment Program) [120]. To accurately and reliably quantify important disparities in AC-related care and support effective improvement initiatives, attention and effort will need to be invested across the full continuum of quality measure development [121], measure endorsement [122], measure selection, and status assignment within value-based payment programs (e.g., required/optional, measure weighting) [123]. The findings of the scoping review may be of utility to such efforts, and the development and implementation of suitable quality measures will likely be of value to future research efforts in this important therapeutic area.

**Table 3:** Research opportunities: Ethnoracial differences in the quality of anticoagulation care

Topic	Research Opportunity
Overall	Electronic medical record systems providing transparency for how race, ethnicity, and language preference are collected from the patient
	Study investigators collecting and reporting data across the major racial categories with perhaps more specificity for Asian ethnicities
	Ethnoracial disparities in the acute care anticoagulation setting and/or using injectable anticoagulants
	Ethnoracial disparities in anticoagulation indications other than AF
Research Question 1: Access to guideline-based anticoagulation therapy	Reasons why non-White patients (especially Black patients) receive anticoagulation therapy for a guideline-based indication or a DOAC as first-line therapy less frequently than White patients and strategies to close this gap
Research Question 2: Quality of anticoagulation therapy management	More contemporary studies of warfarin INR control in various ethnoracial groups
	More studies on potential ethnoracial gaps in access to an anticoagulation management service, appropriate anticoagulant dosing, PST, and PSM
Research Question 3: Clinical outcomes related to anticoagulation therapy	Ethnoracial disparities in clinical outcomes for anticoagulation indications other than AF
Research Question 4: Humanistic/Educational outcomes related to anticoagulation therapy	Ethnoracial disparities in quality of life and patient satisfaction outcomes across all indications for anticoagulation therapy
	Ethnoracial disparities in anticoagulation knowledge, and effectiveness of anticoagulation delivery systems

DOAC = direct oral anticoagulant; INR = international normalized ratio; PSM = patient self-management; PST = patient self-testing; VTE = venous thromboembolism

## Conclusions

Treatment guidelines do not recommend differentiating anticoagulant therapy by ethnoracial group, yet this scoping review of the literature demonstrates consistent directionality in favor of White patients over non-White patients in the domains of access to anticoagulation therapy for guideline-based indications, prescription of preferred anticoagulation therapies, and quality of anticoagulation therapy management. These data should serve as a stimulus for an assessment of current services, implementation of quality improvement measures, and inform future research to make anticoagulation care quality more equitable.

### Supplementary Information

Below is the link to the electronic supplementary material.

Supplementary file1 (DOCX 22 KB)

Supplementary file2 (DOCX 14 KB)

## Data Availability

Data are available on request from the corresponding author.
