# Supplementary material for: Differences in quality of anticoagulation care delivery according to ethnoracial group in the United States: A scoping review
Source: J Thromb Thrombolysis. 2024 May 11;57(6):1076–91. doi: 10.1007/s11239-024-02991-2 (PMC11315726; doi:10.1007/s11239-024-02991-2)
Supplement: Supplementary file 1 — Supplementary file1 (DOCX 22 KB) [file 11239_2024_2991_MOESM1_ESM.docx]

Supplementary Table. Clinical Outcomes Studies

| **First author, publication year** | **Study period** | **Study Design** | **Number of Patients** | **Ethnoracial groups studied** | **Indication** | **Anticoagulants** | **Clinical Outcomes assessed among ≥ 2 ethnoracial groups** | **Statistical Results*** |
| --- | --- | --- | --- | --- | --- | --- | --- | --- |
| Thigpen, 2012 | Not specified | Single-center prospective cohort | 1,260 | White, Black | Not specified | Warfarin | Major bleeding, minor bleeding | Black patients had higher risk of major bleeding (HR 1.67, 1.13-2.48) but a lower risk of minor bleeding compared to White patients (HR 0.76, 0.64-0.90) |
| Moffett, 2013 | 2004-2009 | Multi-center retrospective cohort | 184 | White, Black, Hispanic, Asian, Other | Pediatrics: AF, VTE treatment, mechanical heart valve, stroke, arterial thrombosis, Fontan procedure, pulmonary hypertension | Warfarin | Bleeding readmission | Asian race was a risk factor for warfarin-related bleeding readmission (OR 3.8, 1.5-9.7) |
| Hankey, 2014 | December 18, 2006-June 17, 2009 | Multi-center RCT sub-analysis (ROCKET-AF) | 14,264 | White, Black, Asian, Other | AF | Warfarin, rivaroxaban | ISTH major bleeding (intracranial hemorrhage specifically) | Asian (HR 2.02, 95% CI 1.39-2.94) and Black race (HR 3.25, 1.43-7.41) were independent predictors of ICH |
| Hernandez, 2015 | 2010-2011 | Claims database retrospective cohort | 9,404 | White, Black, Hispanic, Asian, Native American/Alaska Native, Other | AF | Warfarin, dabigatran | ISTH major bleeding, any bleeding | Black patients had higher risk of major bleeding (HR 2.09, 1.68-2.60), and both Black (HR 1.16, 1.01-1.34) and Other patients (HR 1.69, 1.24-2.32) had higher risk of any bleeding vs White patients; Asian patients had lower risk of any bleeding compared to White patients (HR 0.67, 0.47-0.95), no difference among groups for all other comparisons |
| Kabra, 2015 | 2010-2011 | Claims database retrospective cohort | 517,941 | White, Black, Hispanic | AF | Warfarin | Stroke/SE, mortality | **see Supplementary Table 2 |
| DiNisio, 2016 | March 2007-September 2009 and March 2007-March 2011 | Multi-center RCT sub-analysis (EINSTEIN DVT and EINSTEIN PE) | 8,246 | White, Black, Asian, Other | VTE treatment | Warfarin, rivaroxaban, UFH, LMWH, fondaparinux | ISTH major bleeding <3 weeks from VTE, >3 weeks from VTE, total study period | Black patients at higher risk of bleeding in first 3 weeks vs White patients (HR 3.26, 1.15-9.23); no significant difference among groups for the other two outcomes |
| Majeed, 2016 | April 2006-November 2008 | Multi-center RCT subanalysis (RECOVER and RECOVER II) | 5,001 | White, Asian | VTE treatment | Warfarin, dabigatran | ISTH major bleeding, CRNMB, any bleeding | Asian higher risk of major bleeding (HR 2.15, 1.2-3.9) but no difference for major+CRNMB or any bleeding |
| Banala, 2017 | 2013-2014 | Single-center retrospective cohort | 193 | White, Black, Hispanic, Other | Cancer-associated VTE treatment | Any anticoagulant | 30-day, 90-day, and overall PE survival in cancer patients | No significant difference among ethnoracial groups |
| Kobayashi, 2017 | July 2013-June 2015 | Multi-center prospective cohort | 1,847 | White, Black, Hispanic, Asian | Not specified | Warfarin, any DOAC | ISTH major bleeding, specifically ICH | Asian patients had higher rates of ICH (IRR 1.91 (1.61-2.26), no significant difference among other groups |
| Limdi, 2017 | Not specified | Multi-center prospective cohort | 1,326 | White, Black | Not specified | Warfarin | Major bleeding (serious, life-threatening, fatal) | Black patients had a higher rate of major bleeding compared to White patients (IRR 1.38, 1.01-1.89) |
| Kim, 2018 | 2000-2010 | Claims database retrospective cohort | 9,200,000 | White, Black, Hispanic, Asian, Other | AF | Warfarin | Stroke/SE, hospitalizations, mortality, 2007 | Black patients had higher risk of all adverse outcomes (stroke HR 1.42 p<0.0001, mortality HR 1.27 p<0.0001, hospitalization HR 1.15 p<0.0001); there was no difference in any outcomes between Hispanic and White patients; Asian patients had lower risk of mortality (HR 0.85 p=0.0025) and hospitalizations (HR 0.65 p<0.0001) compared to White patients but no difference in stroke; patients of Other race had higher risk of stroke (HR 1.61 (p=0.0018) compared to White patients, no difference in mortality, and lower risk of hospitalizations (HR 0.87 (p=0.0031) |
| Yamashita, 2018 | Through July 5, 2017 | Meta-analysis | 6 studies, 27,023 patients | Asian, non-Asian | VTE treatment | Warfarin, any DOAC | ISTH major bleeding or CRNMB, recurrent VTE or VTE-related mortality | No significant difference among ethnoracial groups |
| Abu-Zeinah, 2019 | 2004-2017 | Single-center retrospective cohort | 84 | White, Black, Asian, Other, Not Available | APS | Warfarin, any DOAC, UFH, LMWH | Recurrent thrombosis (any) during anticoagulation | Black patients higher risk of recurrent thrombosis vs White patients (HR 5.94, 95% CI 1.1-32.1, p=0.039) |
| LaDuke, 2019 | 2012-2017 | Multi-center retrospective cohort | 5,161 | White, Black, Other | Not specified | Warfarin, any DOAC | Mortality or hospice after trauma on anticoagulation | Both Black (OR 0.83, 0.71-0.96) and Other race patients (OR 0.80, 0.65-0.99) had lower odds of mortality or hospice after trauma compared to White patients |
| Schwartz, 2019 | 2011-2017 | Single-center retrospective cohort | 21,648 | White, non-White | AF | Warfarin, any DOAC | Any bleeding | Frequency of any bleeding was higher in non-White patients (122 per 1,000 person-years vs 21.2 per 1,000 person-years, p<0.0001) |
| Addo-Tabiri, 2020 | 2004-2018 | Single-center retrospective cohort | 161 | White, Black, Other | Cancer-associated VTE treatment | Warfarin, DOAC, UFH, LMWH | Recurrent cancer-associated VTE | No significant difference among ethnoracial groups |
| Akhtar, 2020 | January 2015-April 2018 | Single-center retrospective cohort | 326 | White, Black, Hispanic, Asian, Other | AF | Rivaroxaban | ISTH major/CRNMB | No significant difference among ethnoracial groups |
| Doucette, 2020 | 2014-2015 | Single-center retrospective cohort | 379 | White, Black | AF or VTE | Any DOAC | ISTH CRNMB or rivaroxaban in overweight patients | No significant difference among ethnoracial groups |
| Tedla, 2020 | 2011-2017 | Multi-center retrospective cohort | 11,575 | White, non-White | AF | Warfarin, any DOAC | ISTH major bleeding, stroke/SE | DOACs reduced rates of stroke and bleeding in White patients but not in non-White patients (aIRR Whites 0.52, 0.31-0.85, non-Whites 0.71, 0.22-2.31) |
| Aguilar, 2021 | November 2017-May 2018 | Single-center retrospective cohort | 1,087 | White, Hispanic, Other | AF or VTE treatment | Any DOAC | 30-day re-admissions, 1-year mortality | No significant difference among ethnoracial groups |
| Chen, 2021 | 2014-2015 | Claims-database, latent-class analysis | 16,399 | White, Black, Hispanic, Other | AF | Warfarin or any DOAC | Stroke/SE | Black patients at higher risk for stroke/SE vs White patients (HR 1.41, 1.21-1.63) |
| Gu, 2021 | 2013-2018 | Multi-center registry | 1,359,827 | White, Asian | AF | Warfarin, any DOAC | Bleeding requiring hospitalization, stroke/SE, all-cause mortality | No significant difference among ethnoracial groups |
| Cires Drouet, 2022 | January 2016-February 2018 | Single-center retrospective cohort | 1,000 | White, Black | Upper Extremity VTE treatment | Any anticoagulant | Recurrent VTE, ISTH major bleeding | No significant difference among ethnoracial groups |
| Gencer, 2022 | 2008-2010 | Multi-center RCT sub-analysis (ENGAGE AF-TIMI) | 21,104 | Asian, Non-Asian | AF | Warfarin, Edoxaban | Net clinical outcome: stroke/SE, major bleeding, all-cause mortality | Asian patients had significantly more favorable net clinical outcome vs non-Asian patients for both doses of edoxaban vs warfarin (high-dose edoxaban HR 0.75, 0.62-0.92, low-dose edoxaban HR 0.73, 0.60-0.89) |

*all confidence intervals represent 95% confidence interval
AF=atrial fibrillation; CRNMB=clinically relevant nonmajor bleeding; DOAC=direct oral anticoagulant; HR=hazard ratio; ICH=intracranial hemorrhage; IRR=incident rate ratio; ISTH=International Society on Thrombosis and Haemostasis; LMWH=low molecular weight heparin; OR=odds ratio; PE=pulmonary embolism; RCT=randomized controlled trial; SE=systemic embolism; UFH=unfractionated heparin; VTE=venous thromboembolism
