# Supplementary material for: Differences in quality of anticoagulation care delivery according to ethnoracial group in the United States: A scoping review
Source: J Thromb Thrombolysis. 2024 May 11;57(6):1076–91. doi: 10.1007/s11239-024-02991-2 (PMC11315726; doi:10.1007/s11239-024-02991-2)
Supplement: Supplementary file 2 — Supplementary file2 (DOCX 14 KB) [file 11239_2024_2991_MOESM2_ESM.docx]

Supplementary Table 2. Kabra, 2015 Clinical Outcomes Hazard Ratios*

|  | **Age 66-74 years** | **Age 75-84 years** | **Age 85+ years** | **Clinical Outcome Summary** |
| --- | --- | --- | --- | --- |
| Stroke by age-Black vs White | 1.48 (1.33-1.65) | 1.54 (1.41-1.68) | 1.32 (1.20-1.46) | Black patients had higher risk of stroke compared to White patients across all age groups |
| Death by age-Black vs White | 0.91 (0.88-0.94) | 0.98 (0.95-1.01) | 0.93 (0.90-0.96) | Black patients had lower risk of death compared to White patients across all age groups |
| Stroke by age-Hispanic vs White | 1.22 (1.06-1.40) | 1.13 (1.02-1.25) | 1.02 (0.9-1.15) | Hispanic patients had higher risk of stroke compared to White patients across ages 66-84, no difference in patients aged 85+ |
| Death by age-Hispanic vs White | 0.79 (0.75-0.83) | 0.81 (0.78-0.84) | 0.88 (0.85-0.91) | Hispanic patients had lower risk of mortality compared to White patients across all age groups |
|  | | | | |
|  | **CHA_2_DS_2_VASc score 0-4** | **CHA_2_DS_2_VASc score 5-6** | **CHA_2_DS_2_VASc score 7+** | **Clinical Outcome Summary** |
| Stroke by CHA_2_DS_2_VASc score Black vs White | 1.71 (1.51-1.94) | 1.43 (1.32-1.54) | 1.37 (1.24-1.50) | Black patients had higher risk of stroke compared to White patients for any CHA_2_DS_2_VASc score |
| Death by CHA_2_DS_2_VASc score Black vs White | 1.00 (0.96-1.04) | 0.94 (0.92-0.96) | 0.96 (0.93-1.00) | Black patients had lower risk of death compared to White patients for CHA_2_DS_2_VASc score 5+, no difference between groups for CHA_2_DS_2_VASc score 0-4 |
| Stroke by CHA_2_DS_2_VASc score Hispanic vs White | 1.25 (1.07-1.45) | 1.05 (0.95-1.16) | 1.10 (0.98-1.24) | Hispanic patients had higher risk of stroke compared to White patients for CHA_2_DS_2_VASc 0-4, no difference in CHA_2_DS_2_VASc 5+ |
| Death by CHA_2_DS_2_VASc score Hispanic vs White | 0.79 (0.75-0.83) | 0.82 (0.79-0.84) | 0.88 (0.85-0.92) | Hispanic patients had lower risk of death compared to White patients for any CHA_2_DS_2_VASc score |

*all confidence intervals represent 95% confidence interval
CHA_2_DS_2_VASc=stroke risk stratification system where patients receive 1 point for congestive heart failure, 1 point for hypertension, 2 points for age ≥ 75 years, 1 point for diabetes, 2 points for prior stroke, 1 point for vascular disease, and 1 point for female sex category
